# *Pumilio2* regulates synaptic plasticity via translational repression of synaptic receptors in mice

**DOI:** 10.18632/oncotarget.24345

**Published:** 2018-01-30

**Authors:** Hongxin Dong, Mengyi Zhu, Liping Meng, Yan Ding, Ding Yang, Shanshan Zhang, Wenan Qiang, Daniel W. Fisher, Eugene Yujun Xu

**Affiliations:** ^1^ Departments of Psychiatry and Behavioral Sciences, Northwestern University Feinberg School of Medicine, Chicago, IL 60611, USA; ^2^ State Key Laboratory of Reproductive Medicine, Nanjing Medical University, Nanjing, 211166, P. R. China; ^3^ Department of Obstetrics and Gynecology, Northwestern University Feinberg School of Medicine, Chicago, IL 60611, USA; ^4^ Department of Neurology, Northwestern University Feinberg School of Medicine, Chicago, IL 60611, USA

**Keywords:** Pumilio (PUM), RNA binding protein, dendrite, synapse, glutamate receptor 2 (GLUR2)

## Abstract

PUMILIO 2 (PUM2) is a member of Pumilio and FBF (PUF) family, an RNA binding protein family with phylogenetically conserved roles in germ cell development. The Drosophila Pumilio homolog is also required for dendrite morphogenesis and synaptic function via translational control of synaptic proteins, such as glutamate receptors, and recent mammalian studies demonstrated a similar role in neuronal culture with associated motor and memory abnormalities *in vivo*. Importantly, transgenic mice with PUM2 knockout show prominent epileptiform activity, and patients with intractable temporal lobe epilepsy and mice with pilocarpine-induced seizures have decreased neuronal PUM2, possibly leading to further seizure susceptibility. However, how PUM2 influences synaptic function *in vivo* and, subsequently, seizures is not known. We found that PUM2 is highly expressed in the brain, especially in the temporal lobe, and knockout of *Pum2* (*Pum2*^*–/–*^) resulted in significantly increased pyramidal cell dendrite spine and synapse density. In addition, multiple proteins associated with excitatory synaptic function, including glutamate receptor 2 (GLUR2), are up-regulated in *Pum2*^***–/–***^ mice. The expression of GLUR2 protein but not mRNA is increased in the *Pum2*^*–/–*^ mutant hippocampus, *Glur2* transcripts are increased in mutant polysome fractions, and overexpression of PUM2 led to repression of reporter expression containing the 3′Untranslated Region (3′UTR) of *Glur2*, suggesting translation of GLUR2 was increased in the absence of *Pum2*. Overall, these studies provide a molecular mechanism for the increased temporal lobe excitability observed with PUM2 loss and suggest PUM2 might contribute to intractable temporal lobe epilepsy.

## INTRODUCTION

Increasing evidence is implicating synaptic dysfunction in temporal lobe structures, especially the hippocampus, as being causally important to many neuropsychiatric disorders, such as epilepsy, Post Traumatic Stress Disorder (PTSD), schizophrenia, and Alzheimer’s Disease (AD) [1–7]. Though many molecular modifications are likely to account for these disease states, post-transcriptional regulation of local mRNA at synapses has emerged as a key mechanism controlling synaptic plasticity and dendrite morphogenesis in both health and disease [8, 9]. Often, these local post-transcriptional mechanisms rely on RNA-binding proteins (RBP), but detailed accounts of how these proteins affect synaptogenesis are in their infancy.

One important RBP found at excitatory synapses is PUMILIO 2 (PUM2), a member of the Pumilio and FBF (PUF) family, which is evolutionarily conserved from *Drosophila* to mice and humans [10–14]. PUM proteins bind to the 3′ Untranslated region (3′ UTR) of their target mRNAs, likely halting translation until certain synaptic signals are presented [11, 15, 16]. PUM2 binds to specific RNA sequences known as Nanos response elements (NREs), also called Pumilio Binding Element (PBE) [12, 15, 17, 18], and PUM2 may also be highly regulated by the *Noncoding RNA activated by DNA damage (NORAD)* [19, 20].

While many studies have shown that mammalian PUM proteins are important for germ cell formation and differentiation during reproductive system development [11, 21], a growing number of studies indicate that PUM proteins may influence motor and neurological disorders, such as epilepsy [7, 22–25]. In conjunction, PUM homologs have been found in neuronal cells and regulate neuronal homeostasis through affecting dendritic structure, synaptic plasticity, and neuronal excitability in *Drosophila* [26–30] Some of the gene targets of PUM proteins have been identified in the *Drosophila* nervous system, including certain glutamate receptors [30–33].

Importantly, recent studies indicate that *Pum2* is highly expressed in the mammalian brain [23, 34], and the role of PUM2 in mammalian neuronal regulation have been suggested by *in vitro* studies. In particular, reduction of the *Pum2* expression via shRNA accelerates dendrite outgrowth and arborization in rodent primary hippocampal neurons [13]. In addition, PUM2 is a component of dendritically localized ribonucleoparticles (RNPs), suggesting a role of PUM2 in the neuronal response to cellular stress [13, 35]. Mice with *Pum2* knockout exhibit hyperactivity, spontaneous spike-wave discharges, and a reduced seizure threshold to chemoconvulsants [22]. Recently PUM2 was shown to regulate neurogenesis together with PUM1, further establishing important roles of PUM proteins in mammalian nervous system [36]. Interestingly, reduced PUM2 was found in the neocortex of patients with drug-refractory Temporal Lobe Epilepsy (TLE) as well rats given seizures through pilocarpine injections [37]. Additionally, pilocarpine-treated rats that developed status epilepticus still had reduced hippocampal and cortical PUM2 up to 60d later, a time point where spontaneous epileptiform activity develops and closely models TLE pathogenesis [37]. These results suggest that decreased PUM2 may promote increased excitability that leads to epileptogenesis [7].

In this study, we aimed to investigate how PUM2 influences dendritic morphology, synaptic density and synaptic proteins in the cortex and hippocampus of mice. In particular, we focused on how PUM2 may influence excitatory transmission through glutamatergic signaling pathways by investigating PUM2’s regulation of *Glur2* mRNA translation. Our study provides the first direct evidence for a post-transcriptional role of PUM2 in regulating *Glur2* translation and excitatory synapse morphogenesis in mammals.

## RESULTS

### *Pum2* is highly expressed in the temporal lobe

**Figure 1:**
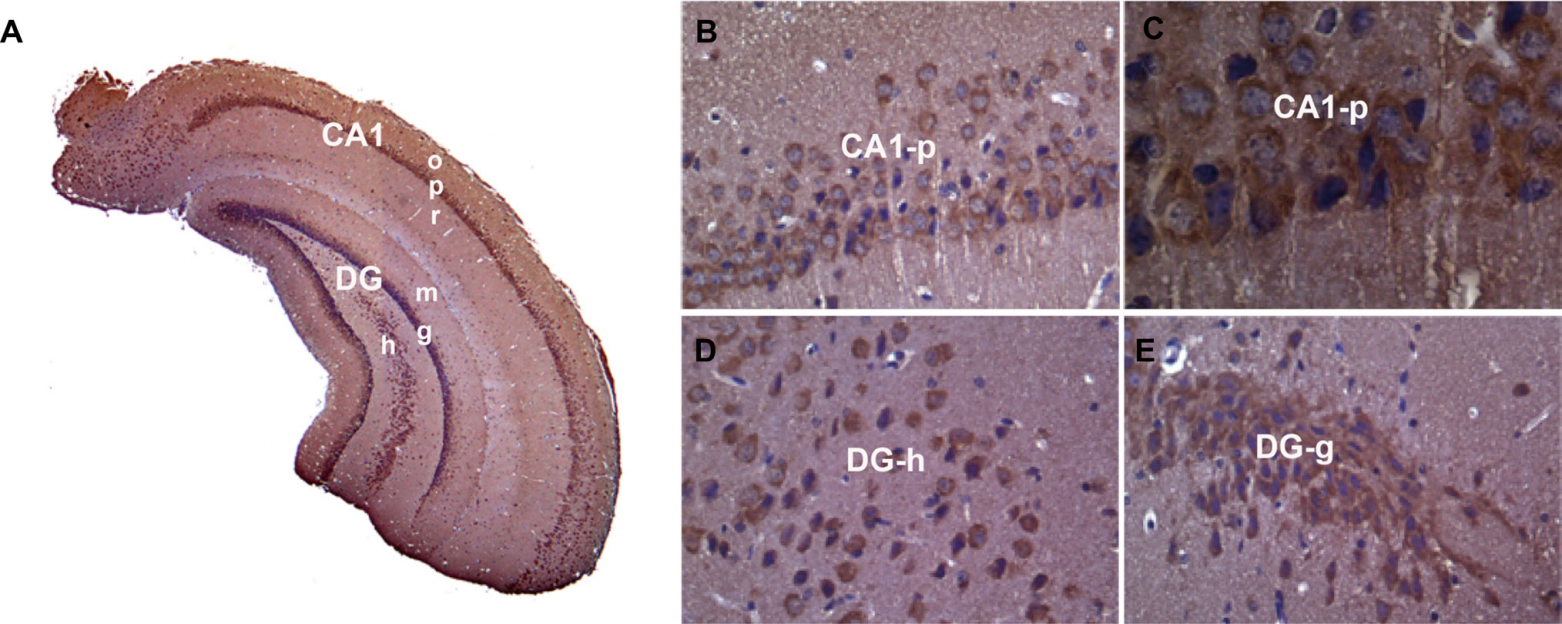
PUM2 protein is highly expressed in the mouse hippocampus Immunohistochemistry using PUM2 antibody showed that PUM2 proteins are enriched in CA1-3 and dentate gyrus (**A**). At higher magnification of subregions, PUM2 signals (brown color) were located mainly in the cytoplasm and projections of neuronal cells (**B**–**E**), The structures with blue color staining are nuclei. CA1: hippocampal CA1 area; DG: Dentate gyrus; o: stratum oriens; p: striatum pyramidale; r: striatum radiatum; m: dentate molecular layer; g: granular cell layer; h: hilus proper.

*Pum2*^*XE772*^ mutant mice carry the *LacZ* gene under the endogenous *Pum2* promoter. Hence, X-gal staining can reveal the expression pattern of *Pum2* at the cellular level. X-gal staining demonstrated that *Pum2* was expressed in many brain areas, including the cortex, amygdala, thalamus, hypothalamus, and cerebellum, with the highest expression in the temporal lobe, especially the hippocampus [22] (Supplementary Figure 1). Although the *Pum2*-driven *LacZ* reporter method nicely reflects the expression pattern of *Pum2* transcription in the brain, protein localization may not be the same as its mRNA expression, and this method is unable to resolve the subcellular localization of PUM2. Therefore, we conducted immunohistochemical staining to determine PUM2 expression in wild type mice, and demonstrated that PUM2 protein closely follow X-gal staining with high expression of PUM2 in the hippocampus, mainly localized to the cytoplasm and dendritic projections of pyramidal cells. (Figure 1).

### *Pum2* knockout affects hippocampal spine and synapse densities *in vivo*

**Figure 2:**
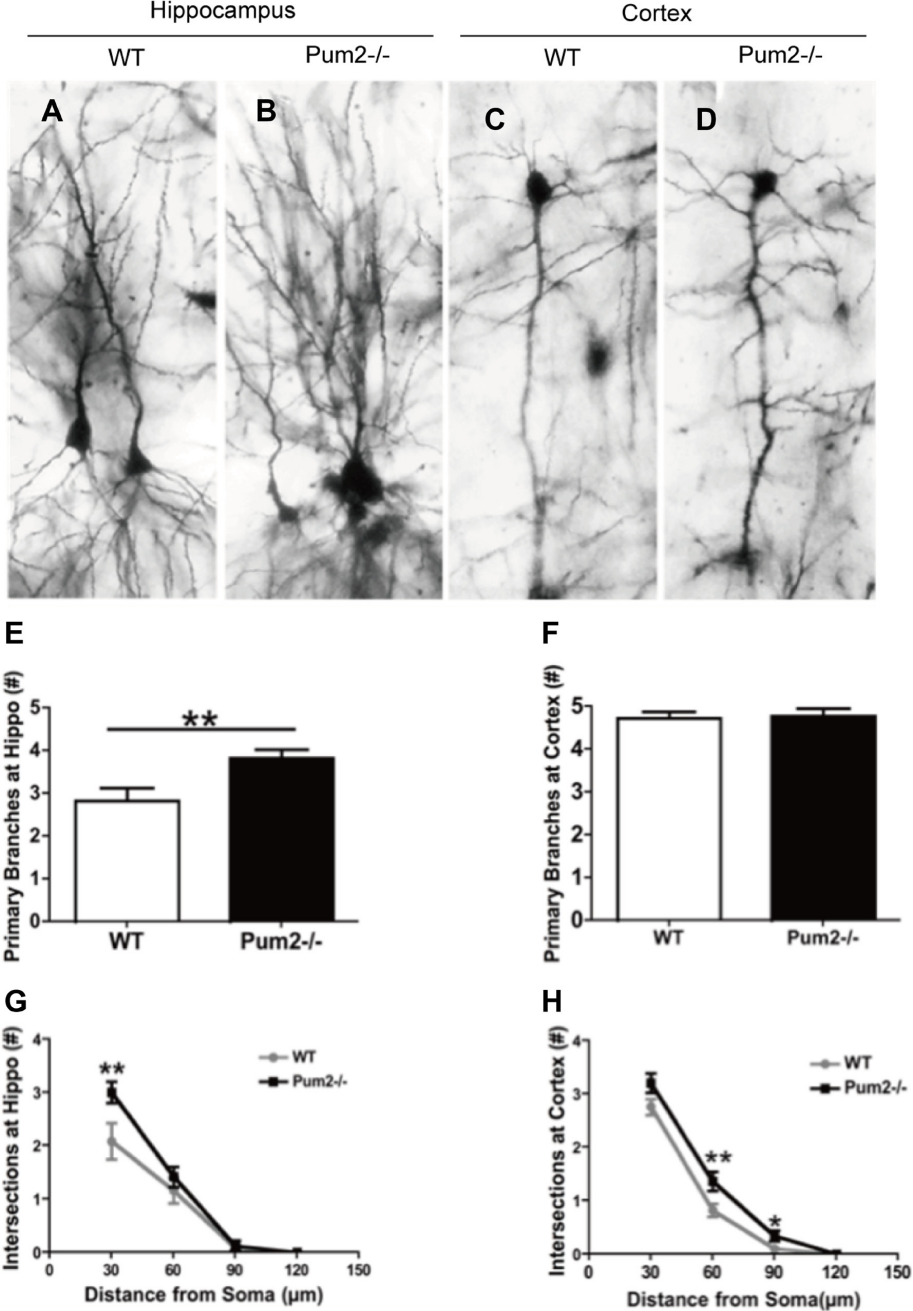
Dendritic branches of *Pum2*^*–/–*^ and WT mice via Golgi staining Representative pyramidal neurons in the CA1 area of the hippocampus (**A**: WT, l **B**:*Pum2*^*–/–*^) and the cortex (**C**:WT and **D**: *Pum2*^*–/–*^). There is a significant increase in primary dendritic branching in the *Pum2*^*–/–*^ mice as compared to WT in the hippocampus (**E**) but not in the cortex (**F**). Additional measurements of the dendritic branch intersections using Sholl analysis in consecutive 30, 60, 90, and 120 µm concentric bands radiating from the center of the soma indicated a significant difference between *Pum2*^*–/–*^ and WT mice (**G** and **H**). ^*^means *p* < 0.05 and ^**^means *p* < 0.01. Data represent as mean ± SEM.

**Figure 3:**
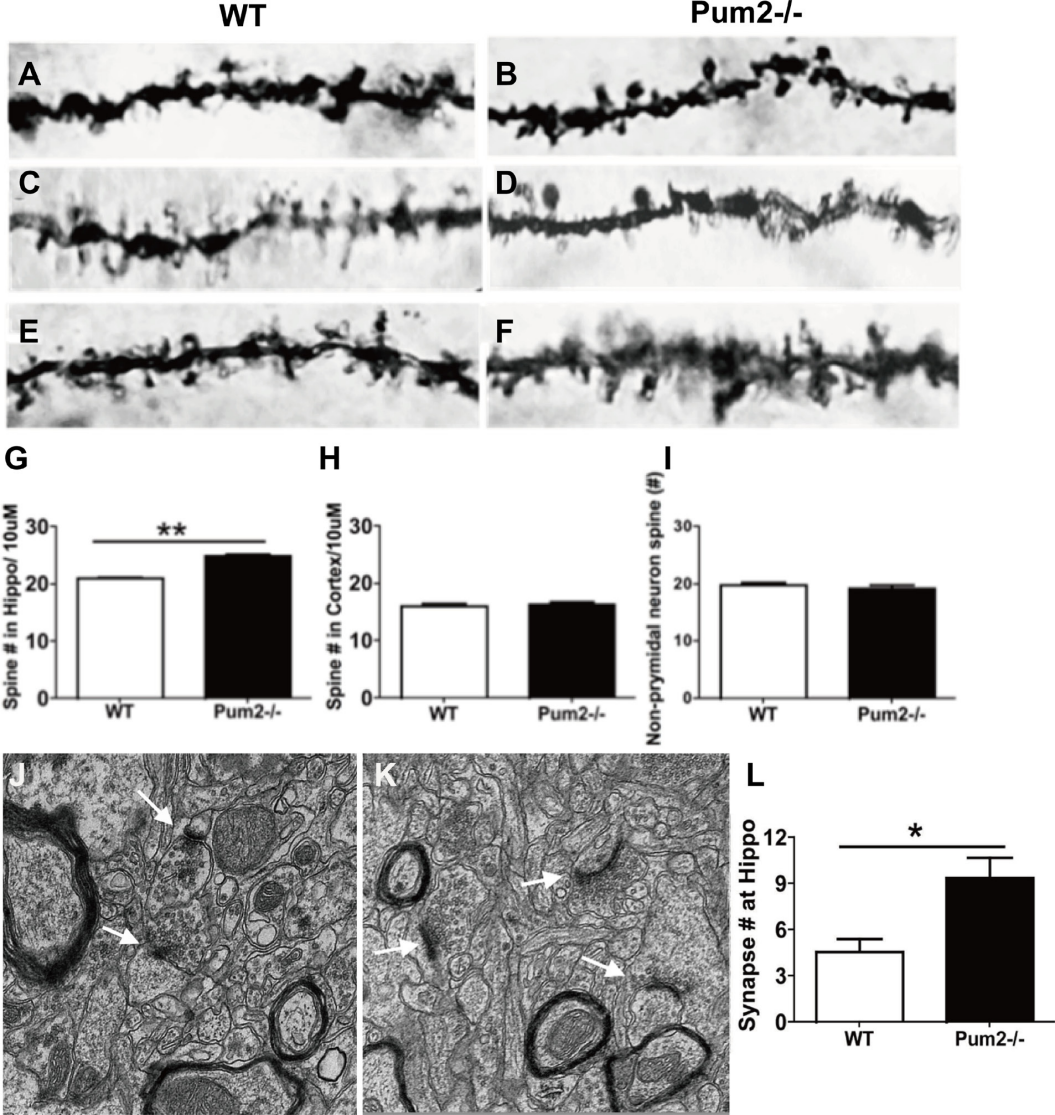
Spine density of *Pum2*^*–/–*^ and WT mice by Golgi staining Spine density significantly increased in the pyramidal cells within the CA1 area of hippocampus (**A**, **B**, **G**) but not in layer V of the frontal cortex (**C**, **D**, **H**) and non-pyramidal cells within the CA1 area of hippocampus (**E**, **F**, **I**). (**J**) Electron microscopy (EM) quantification of synapses in the stratum radiatum of the CA1. The representative images demonstrated the ultrastructure of synapses in the stratum radiatum in WT mice (J) and *Pum2*^*–/–*^ (**K**) (white arrows indicate synapses). Quantification of synapse number revealed significantly higher number in *Pum2*^*–/–*^ than WT mice (**L**). ^*^means *p* < 0.05. Data represent as mean ± SEM.

We next investigated how loss of *Pum2* affected dendritic spine and synaptic density of pyramidal cells in the cortex and hippocampus of wild-type (WT) and *Pum2*^–/–^ mice using Golgi Staining. Pyramidal cells in the CA1 layer of the *Pum2*^*–/–*^ hippocampus displayed significantly increased primary dendritic branching in apical dendrites as compared to age-matched WT mice (*Pum2*^*–/–*^ 3.83 ± 0.20 vs. WT 2.83 ± 0.30; *P* < 0.01) (Figure 2A, 2B, 2E); however, these increases were restricted to the hippocampus and were not seen in the cortex (*Pum2*^*–/–*^ 4.77 ± 0.18 vs. WT 4.72 ± 0.15; *P* > 0.05) (Figure 2C, 2D, 2F). Primary dendritic branch intersections were also quantified using Sholl analysis in consecutive 30, 60, 90, and 120 µm concentric bands radiating from the center of the soma. Similar to primary branch points, dendritic branch intersections within the 30µm band were significantly increased in the *Pum2*^*–/–*^ hippocampus (*Pum2*^*–/–*^ 3.83 ± 0.20 vs. WT 2.8 ± 0.30; *P* < 0.01) (Figure 2G) but not in the frontal cortex (*Pum2*^*–/–*^ 4.78 ± 0.18 vs. WT 4.7 ± 0.15, *P* > 0.05) (Figure 2H) as compared to the same areas of WT mice. Interestingly, in consecutive 60 mm and 90 mm bands, dendritic branch intersections were significantly increased in the frontal cortex of *Pum2*^*–/–*^ mice (60 mm: *Pum2*^*–/–*^ 1.37 ± 0.18 vs. WT 0.82 ± 0.12, *P* < 0.01; 90 mm: *Pum2*^*–/–*^ 0.34 ± 0.09 vs. WT 0.10 ± 0.05, *P* < 0.05,) but not the hippocampus at 60 and 90 mm (60 mm: *Pum2*^*–/–*^ 1.4 ± 0.19 vs. WT 1.17 ± 0.24, *P* = 0.44; 90 mm: *Pum2*^*–/–*^ 0.13 ± 0.09 vs. WT 0.083 ± 0.08, *P* > 0.05) compared to WT mice. Golgi staining also revealed significant increases in average spine density in CA1 pyramidal cells in *Pum2*^*–/–*^ mice (*Pum2*^*–/–*^ 24.86 ± 0.40 vs. WT 20.97 ± 0.26; *P* < 0.01) (Figure 3A, 3B, 3G) but not in layer IV of the frontal cortex (*Pum2*^*–/–*^ 16.44 ± 0.36 vs. WT 16.10 ± 0.35; *P* > 0.05) (Figure 3C, 3D, 3H) or in CA1 interneurons (Figure 3E, 3F, 3I).

Given the consistent changes in the proximal dendrites of the CA1, we analyzed the synaptic ultrastructure of these cells at the stratum radiatum using electron microscopy (EM). We found that *Pum2*^*–/–*^ mice have significantly higher synaptic densities as compared to WT mice (*Pum2*^*–/–*^ 9.40 ± 1.3 vs. WT 4.6 ± 0.81, *P* < 0.05), and interestingly, we found increased length and width of most postsynaptic density projections in the asymmetric but not symmetric synapses in *Pum2*^*–/–*^ mice as compared to WT mice (Figure 3J, 3K, 3L). In total, these data further indicate that PUM2 regulates synaptic morphology in pyramidal neurons, and loss of PUM2 led to increased asymmetric but not symmetric synaptogenesis, which may predispose these mice to increased excitability through elevated synaptic transmission at excitatory synapses while inhibitory synapses remain unchanged.

### *Pum2* affects excitatory synapse proteins in the hippocampus

*Pum2* clearly affects excitatory synaptic number and morphology in the hippocampus, suggesting synaptic machinery at excitatory synapses may be affected. Therefore, we measured several proteins located in the post-synaptic density that are linked to synaptic plasticity, particularly the glutamate receptors NR2A, NR2B, and GLUR2 (AMPA), phospho-cAMKII, an important member of the calcium/calmodulin-activated protein kinase family, and PSD95, an important post-synaptic scaffolding protein located at excitatory synapses. In addition, we quantified synaptophysin, a presynaptic protein, to determine if PUM2 has trans-synaptic effects. We found that GLUR2 and phospho-CAMK2 protein levels were significantly increased in the hippocampus of *Pum2*^*–/–*^ mice (GLUR2: *Pum2*^*–/–*^ 0.79 ± 0.03 vs. WT 0.38 ± 0.03, *P* < 0.05; phospho-cAMK2: *Pum2*^*–/–*^ 0.68 ± 0.002 vs. WT 0.38 ± 0.05, *P* < 0.05) (Supplementary Figure 2), however, NR2A and NR2B levels were similarly expressed in *Pum2*^*–/–*^ and wild-type mice (NR2a: *Pum2*^*–/–*^ 0.47 ± 0.09 vs. WT 0.38 ± 0.028, *P* > 0.05; NR2b: *Pum2*^*–/–*^ 0.23 ± 0.00004 vs. WT 0.25 ± 0.01, *P* > 0.05), suggesting PUM2 affects LTP-associated proteins downstream of NMDAR signaling (Supplementary Figure 2). In addition, we found PSD95 was also significantly increased in the hippocampus of *Pum2*^*–/–*^ mice (*Pum2*^*–/–*^ 0.84 ± 0.03 vs. WT 0.46 ± 0.061, *P* < 0.05). Importantly, the absence of PUM2 did not lead to significant changes in synaptophysin levels (*Pum2*^*–/–*^ 1.40 ± 0.23 vs. WT 1.49 ± 0.20, *P* < 0.01), consistent with the postsynaptic function of PUM2. This data suggests that in conjunction with morphological changes in pro-excitatory signaling at the synaptic level, important post-synaptic, LTP-associated proteins were also up-regulated with loss of PUM2.

### GLUR2 protein but not mRNA expression is increased in the absence of *Pum2*

**Figure 4:**
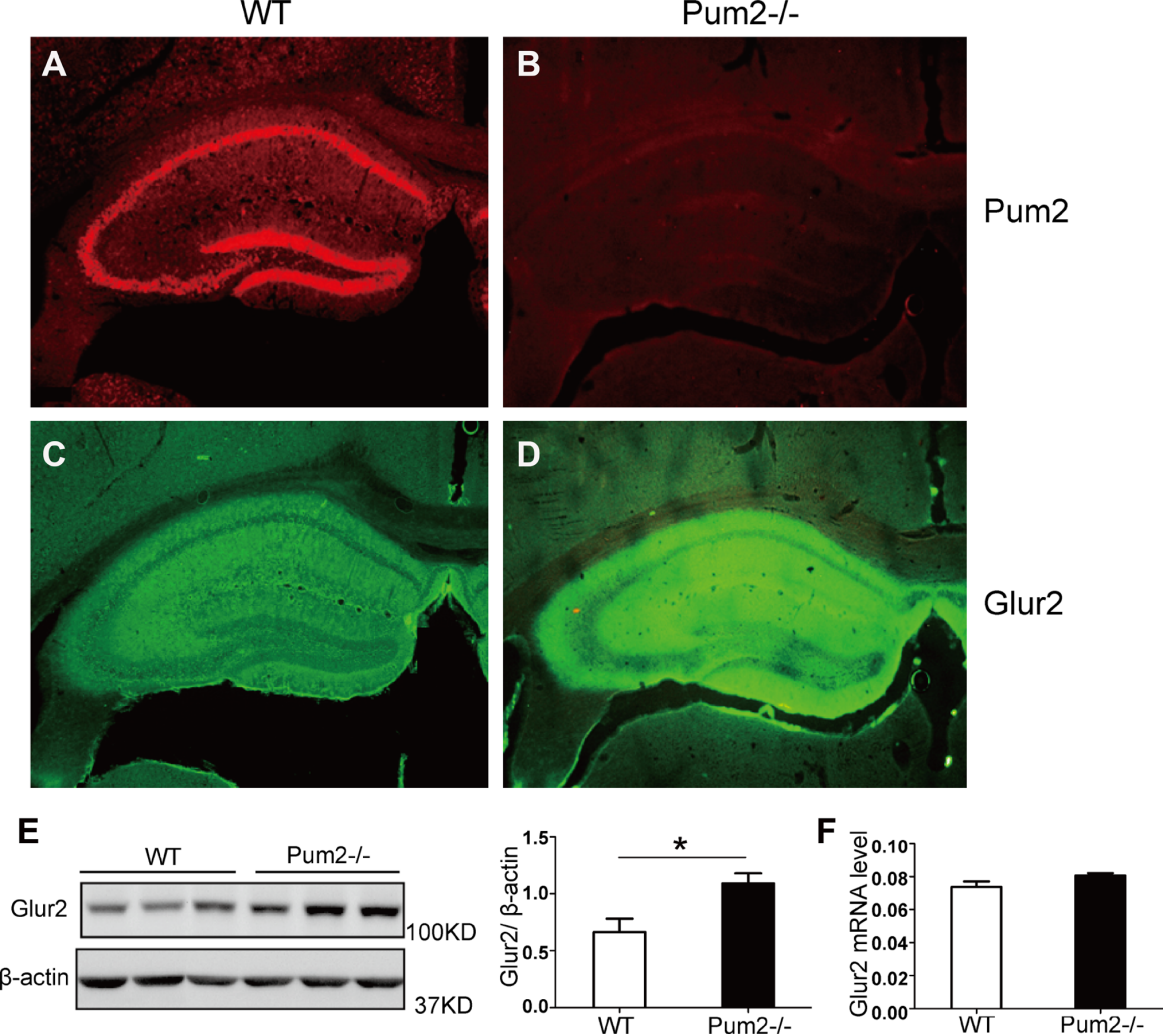
GLUR2 protein level but not mRNA was increased in the absence of *Pum2* (**A**–**D**) Immunofluorescent staining showed that GLUR2 signaling is much stronger in the mutant hippocampus (*n* = 3). (**E)** Western Blot analysis showed that GluR2 protein levels were significantly higher in the hippocampus of *Pum2* mutant mice as compared to WT mice, as analyzed by the relative signal intensity of western blots from three different animals. Mean ± SEM, ^*^p < 0.05. (**F)** mRNA expression of *Glur2* is not different between wildtype and *Pum2*^*–/–*^ mutant hippocampus, suggesting that the increased GLUR2 protein in *Pum2*^*–/–*^ mutant likely resulted from posttranscriptional regulation via PUM2 (*n* = 3 for each genotype). ^*^means *p* < 0.05. Data represent as mean ± SEM.

As GLUR2 was up-regulated in the *Pum2*^–/–^ hippocampus, and previous studies indicate that Drosophila Pumilio protein regulates translation of the Drosophila Glur2 homolog, *Glur2a* [33], we determined whether *Glur2* mRNA in mice is also directly targeted by PUM2. Using immunofluorescence double labeling, we found substantial PUM2 and GLUR2 overlapping expression in the hippocampus. (Figure 4A–4D), with GLUR2 being significantly increased (Figure 4B, 4D, 4E) in *Pum2*^*–/–*^ mice compared to WT. However, there was no significant difference in the total amount of *Glur2* mRNA (Figure 4F), suggesting a post-transcriptional role for PUM2 in regulating GLUR2.

**Figure 5:**
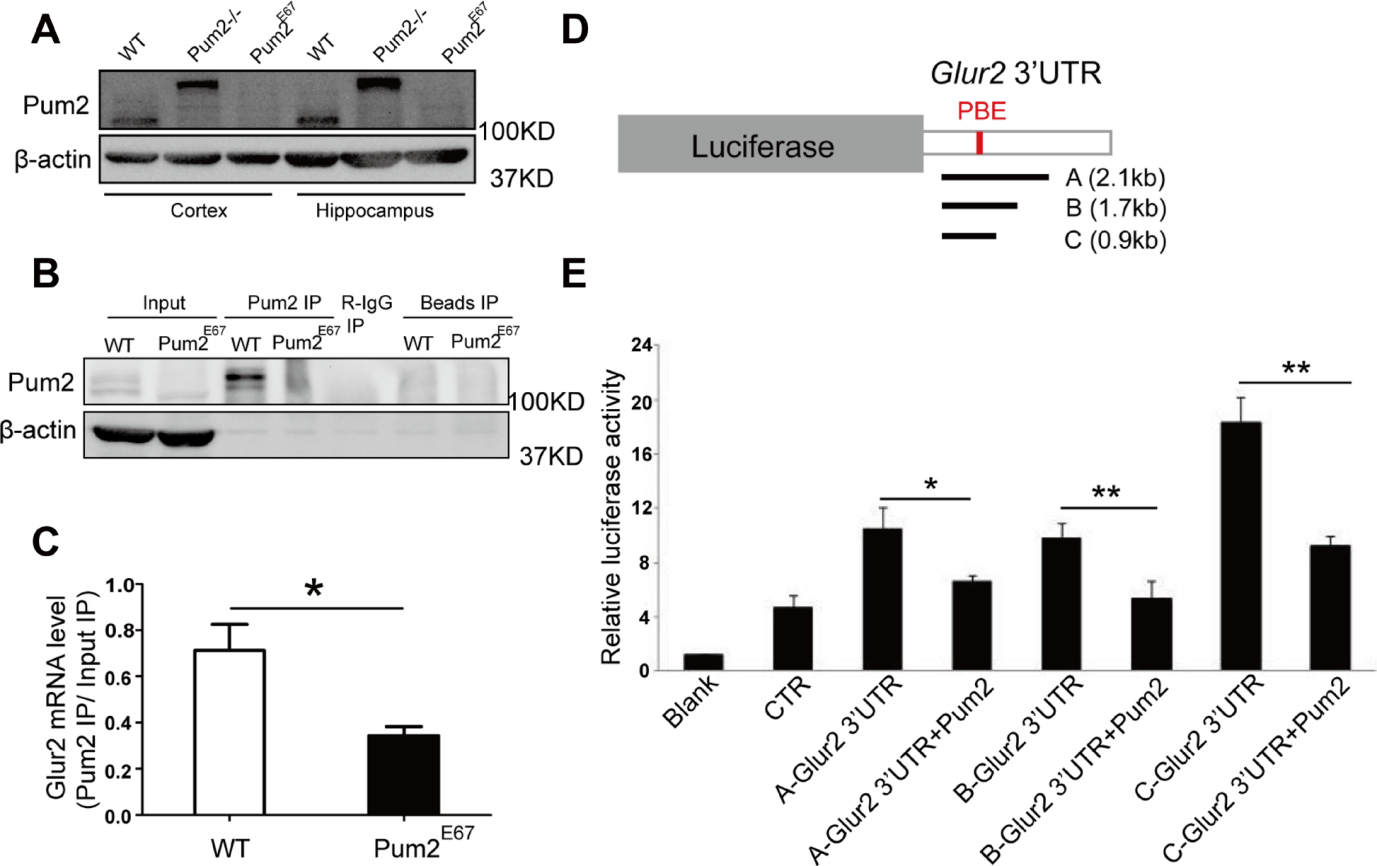
*Glur2* mRNA is associated with PUM2, and PUM2 repressed reporter expression carrying *Glur2* 3′UTR (**A)** PUM2 proteins are completely removed in *Pum2*^*E67*^ cortex and hippocampus. *Pum2*^*–/–*^ does not have the full-length wildtype PUM2 proteins but still produced a bigger fusion protein. (**B)** Western blot of RNA immunoprecipitation experiment revealed that PUM2 proteins could be pulled down in PUM2 immunoprecipitates from the hippocampus (6 animals were used for each genotype). (**C)** *Glur2* mRNA is significantly enriched in PUM2 pull-down, supporting *Glur2* mRNA as a potential PUM2 target. *Glur2* mRNA levels in the pulldown and input were relative to β-actin, and the enrichment of *Glur2* mRNA in the pulldown was based on the *Glur2* mRNA enrichment of pull-down over input. (**D)** Three fragments corresponding to areas of the 3′ UTR containing putative PUM binding motifs were cloned into dual luciferase reporter constructs. (**E)** PUM2 represses translation via the 3′UTR of *Glur2* as shown in dual luciferase assays. Luciferase signals were significantly reduced for all three constructed plasmids carrying different lengths of *Glur2* 3′ UTRs.

**Figure 6:**
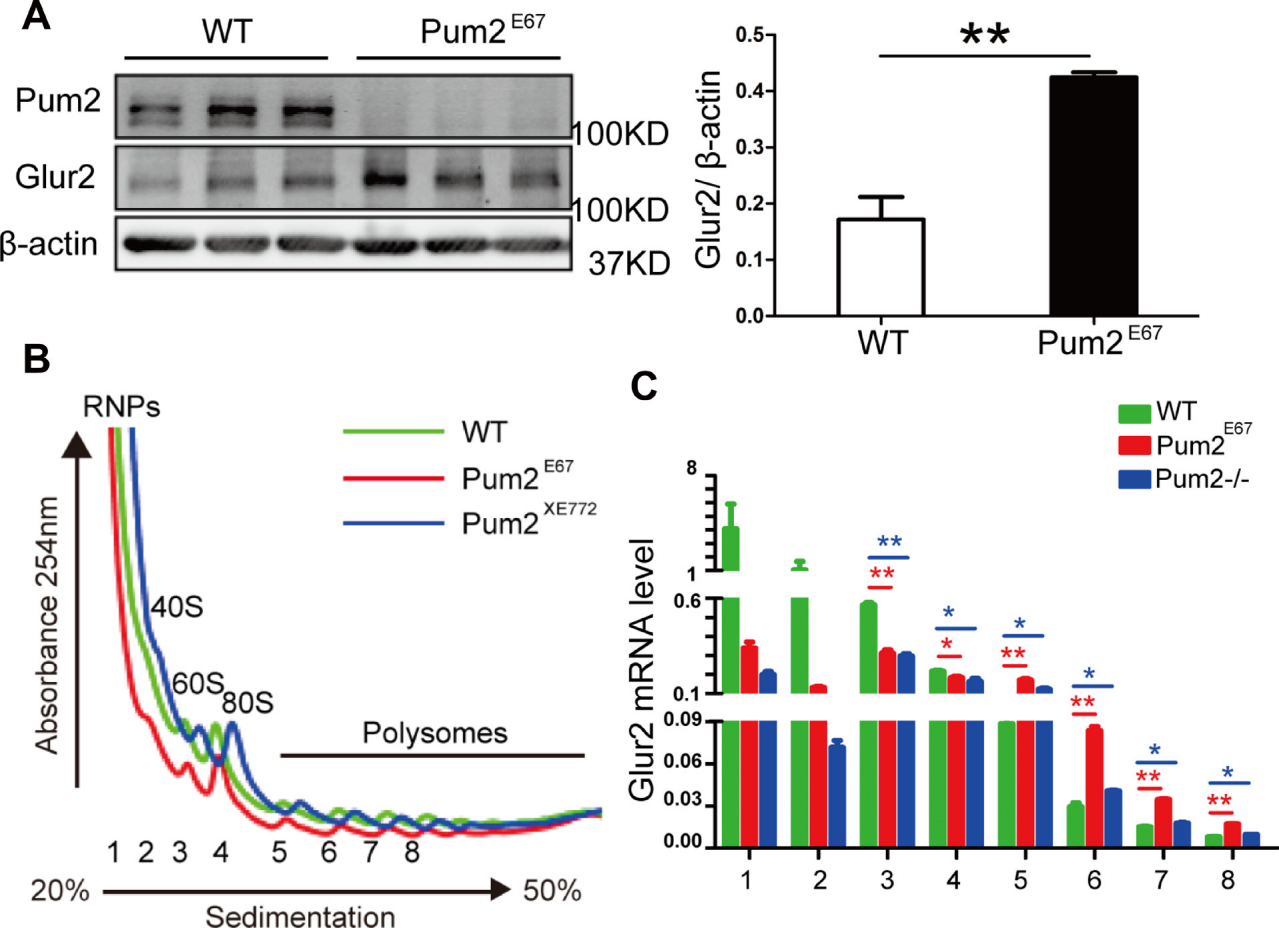
Hippocampal GLUR2 protein translation is significantly up-regulated in two separate loss-of-function *Pum2* mutants (**A**) Hippocampal GLUR2 protein levels were significantly higher in another *Pum2*^*–/–*^ mutant, *Pum2*^*E67*^, while PUM2 protein is completely absent in the mutant hippocampus. On the left is the Western Blot analysis from three pairs of mice, on the right is the quantification of western blot signal intensity (3 animals for each genotype were used). (**B)** Overlay of polysome profiles from wild type (green solid line) and both *Pum2*^*–/–*^ mutants (red and blue solid lines). Tissues from six animals per genotype were used for the experiment. (**C**) qRT-PCR analysis of *Glur2* mRNA from separated fractions showed significantly increased *Glur2* mRNAs in multiple polysome fractions (5, 6, 7 and 8) but not in the free RNP fraction (1). Fractions 2, 3 and 4 correspond to 40 S, 60 S and 80 S fractions. *n* = 6 for each genotype. The *Glur2* mRNA expression data was relative to beta-actin expression from each fraction. ^*^means *p* < 0.05 and ^**^ means *p* < 0.01. Data represent as mean ± SEM.

These results were further confirmed in another independently generated *Pum2* mutant, *Pum2*^*E67*^ mice, as western blot analysis demonstrated that hippocampal GLUR2 protein is up-regulated in absence of *Pum2* (Figure 5A and 6A). Given that the increased number of synapses in *Pum2* mutant tissues may also contribute to the increased GLUR2 protein level, we decided to determine if *Glur2* translation is specifically increased in the mutants and hence contribute to the increase in GLUR2 protein.

### *Glur2* transcripts were associated with PUM2 protein complex in the hippocampus

As the regulatory role of PUM2 at cellular level is likely dependent on its ability to bind RNA, we determined if *Glur2* mRNA is bound by PUM2. Indeed, immunoprecipitation with antibodies specific for PUM2 from hippocampal extracts demonstrated significant binding of PUM2 to *Glur2* in WT but not in *Pum2* knockout mutant (*Pum2*^*E67*^ mice) (Figure 5B and 5C), whereas non-target controls showed no difference between WT and Pum2 mutant RIP (data not shown). In line with this empirical data, a bioinformatic examination of the *Glur2* reference sequence (NM_013540) revealed two PUM binding elements (PBE) in the 3′UTR of *Glur2* (Supplementary Figure 3). These PBE sequences were highly conserved from fish to humans, though stricter conservation was seen for the first PBE than the second (Supplementary Figure 3).

### PUM2 repressed hippocampal *Glur2* translation through its 3′UTR

To determine if PUM2 regulates the expression of *Glur2* through the 3′UTR of *Glur2,* we constructed a dual luciferase reporter construct carrying different lengths of the *Glur2* 3′UTR and measured the fluorescence of both reporters in the presence and absence of PUM2. As expected, the ratio of Firefly luciferase, which had the *Glur2* 3′UTR, to the Renilla luciferase, which was the internal control, significantly decreased when PUM2 was expressed (Figure 5D and 5E), demonstrating that PUM2 likely inhibits *Glur2* translation via the 3′UTR of *Glur2* mRNA. To our surprise, mutations in the two conserved PBEs failed to release PUM2-mediated translational repression (Supplementary Figure 3C), suggesting a mechanism involving sequence outside the two conserved PBEs.

Given that GLUR2 is increased in both *Pum2* mutant mice brains (*Pum2*^*XE772*^ (*Pum2*^*–/–*^) and *Pum2*^*E67*^) (Figures 4E and 6A), and that *in vitro* assays suggest a regulatory role for PUM2 at the 3′ UTR of *Glur2,* we decided to investigate how PUM2 regulates GLUR2 protein expression *in vivo*. Accordingly, we performed polysome analysis on *Glur2* translation in the hippocampus from both our *Pum2* mutants (*Pum2*^*–/–*^ and *Pum2*^*E67*^) and found that *Glur2* transcripts are highly enriched in the polysome fractions but not in RNP fractions of both mutants compared to wildtype controls (Figure 6B and 6C), supporting translational repression of *Glur2* by PUM2 through sequestration of *Glur2* in RNPs and permissive translation of these transcripts with PUM2 ablation.

## DISCUSSION

In this study, we have shown that PUM2 has a dramatic effect on excitatory synapses in pyramidal cells, especially in the hippocampus, at the structural and molecular levels. In particular, loss of PUM2 increases the dendritic branching, spines and synapse density in pyramidal cells, especially in the proximal, apical dendrites of CA1 pyramidal cells. In conjunction, PUM2 regulates the expression of many proteins that are normally up-regulated with post-synaptic mediated LTP but does not affect the upstream NMDARs, perhaps indicating a central role in modifying synaptic strength in response to plasticity-inducing stimuli. Finally, we showed that PUM2 directly regulates *Glur2* through binding to the transcript’s 3′ UTR and sequestering it in RNPs, thereby repressing *Glur2* expression and likely putting an important check on post-synaptic excitation. In total, our results demonstrate a plausible structural and molecular mechanism whereby PUM2 constrains aberrant excitation and plasticity in pyramidal cells and likely explains its dynamic role in epileptogenesis.

Since the discovery of *Drosophila* PUM gene in 1987, an increasing number of its homologs have been identified in other species with varying physiological functions [10, 11, 14]. PUM is necessary for embryonic patterning and is involved in multiple stages of germ cell development in *Drosophila*, and its extraordinary conservation across most, if not all, invertebrate and vertebrate species suggest a critical function in most eukaryotes [30, 38–41]. While it has been hypothesized that the ancestral function of PUM is in regulating germline stem cell proliferation and differentiation, but it’s clear that phylogenetically higher organisms have developed additional regulatory roles for these proteins [11, 14, 42]. In line with this, data suggest that PUM is required for synaptic growth, plasticity, and memory in *Drosophila* [32, 33, 43]. In mammals, PUM2 also appears to be involved in synaptic plasticity and neuronal activities [7, 13, 22, 35], suggesting that PUF family proteins may also have an integral role in neuronal function and circuit homeostasis. Consistent with this hypothesis, our data presented here depict PUM2 as an important regulator of excitatory transmission through dynamic control of synapse structure and number as well as the molecular machinery involved in synaptic strengthening.

Previously, we reported that the *Pum2* is expressed in most brain regions in mice [23], and we’ve further confirmed this expression pattern with more detailed brain subregion distribution, demonstrating particular high expression of PUM2 in important temporal lobe areas, including the cortex, amygdala, and hippocampus, as well as noticeable expression in the thalamus, hypothalamus, and cerebellum (Supplementary Figure 1). Our PUM2 immunohistochemical staining further confirms that PUM2 protein is expressed similarly in these regions.

Intriguingly, *Pum2*^–/–^ mutant mice exhibited significantly greater outgrowth of primary dendrites, dendritic arborization, spine density, and synaptic numbers in pyramidal neurons in the hippocampus but not in the cortex, suggesting that the impact of the dendritic morphology may be restricted to those areas. In addition, most of these increases in synaptic morphology were most prominent in the proximal dendrites of CA1 pyramidal cells, though the functional significance of this region-dependent regulation will still need closer investigation. These results establish an important role of PUM2 in dendritic morphogenesis and synapse function in a subregion-dependent manner not yet realized by previous *in vitro* studies [13].

Previous studies in *Drosophila* show that PUM is involved in long-term memory and alters both pre- and post-synaptic mechanisms of excitability at the neuromuscular junction, partly through regulating *Glur2a* and *eIF-4e* [33, 43]. While these experiments in *flies* are indicative of the role of PUM2 in memory, recent mammalian studies by our group (data not show) and others (Siemen *et al.* 2011) have not detected similar changes in hippocampus-dependent memory upon PUM2 ablation, making the role of PUM2 in mammals less clear. However, PUM2 ablation in mice leads to spontaneous epileptiform activity in the form of aberrant spike-wave discharges and a decreased seizure threshold to the seizure-inducing drug pentylenetetrazole [7, 22]. In line with this, patients with medically refractory TLE have reduced neocortical PUM2, and rats treated with pilocarpine leading to status epilepticus and subsequent spontaneous seizures days to weeks later also demonstrate a similar reduction in cortical and hippocampal PUM2 [37]. The pyramidal neurons of the hippocampus are the principal glutamatergic neurons [44–49], and the associated neuropil accounts for the most dendritic spines found in the hippocampal CA regions [50–54]. Impaired glutamatergic neurotransmission at the pre- and post-synaptic levels has been suggested to cause epileptic seizures, which are prominent in *Pum2*^–/–^ mice observed in both our group and others [55–62]. It is well-established that an initial severe seizure may predispose an individual to future seizures – and certainly chemoconvulsant-induced status epilepticus with subsequent spontaneous seizures in rodents is a strong model of this form of epileptogenesis - but the multifaceted mechanism behind this phenomenon is still a hot topic for epilepsy researchers. Therefore, it’s particularly intriguing that seizure-induced loss of PUM2 persists in pilocarpine-treated rats well into the period where spontaneous seizures arise. The increased GLUR2 and phospho-CAMK2 expression in *Pum2*^*–/–*^ mice found in our study, along with PUM2’s impact on dendritic morphogenesis and density within the hippocampus, may support a mechanism where translational de-repression of key postsynaptic proteins leads to increased excitation of pyramidal cells within the temporal lobe, leading to favorable conditions for seizure development in *Pum2*^*–/–*^ mice. Increased number of synapses in the mutant may also contribute to the increased level of synaptic proteins such as GLUR2, such contribution, however, may be very limited as other synaptic proteins such as synaptophysin (Supplementary Figure 2) did not exhibit similar increase. Therefore, mammalian PUM2 may have a major role in the homeostatic regulation of excitatory signaling that is dysfunctional in TLE. Accordingly, an interesting avenue for future research will be to see if increased activation of the PUM2 regulatory pathway could represent a therapeutic strategy to reverse epileptogenic mechanisms in medically refractory patients.

PUM proteins are known to regulate target gene expression by binding the PBE motif on the 3′UTR of their target transcripts. Hence it is intriguing that mutated PBE in *Glur2* 3′UTR did not release the repression of PUM2 on luciferase expression, raising the possibility that PUM2 repressed *Glur2* translation independent of these two consensus PBE sites. One possibility is that PUM2 regulate Glur2 translation indirectly, via other proteins or miRNAs. Consistent with this possibility, *Glur2* was not identified as a direct target by PUM2 iClip [36] and PUM proteins were reported to regulate miRNA-mediated repression of their common targets [63]. Alternatively PUM2 bind to other sites slightly different from concensus PBE sites, further characterization of the smallest region (0.9 kb) subject to repression could distinguish the two possibilities.

Apart from epilepsy, it is reasonable to hypothesize that alterations in PUM2 may be associated with certain neuropsychiatric disorders that are caused by disruption of excitatory synapse homeostasis, especially those specific to hyperactivity or neurodegeneration. In support of this, PUM2 has been found to interact with multiple genes encoding functional proteins that are highly implicated in Alzheimer’s Disease, including amyloid precursor protein (APP), tau protein, and elF-4E [13, 32, 33]. Furthermore, abnormalities in synaptic pruning are thought to be a significant pathological mechanism leading to both schizophrenia and major depressive disorder [64, 65], and PUM2’s contribution to pruning during puberty is considered an important component of the transition from young to mature brain structures during normal development [66]. Our study and others suggest that PUM2 may have a broad effect in the brain function and may directly associate with multiple neuropsychiatric disorders; however, a closer look at the relationship between PUM2 and these specific neuropsychiatric disorders is warranted in future studies.

In summary, our study provides strong evidence that PUM2 plays a significant role in the regulation of synaptic structure, suggesting that neuronal function of PUF proteins may also be highly conserved in addition to their germline function. Future studies of investigating PUM2 during synaptic formation and procession in pathological conditions, such as epilepsy and schizophrenia, with a particular focus on glutamatergic signaling and resultant plasticity, could reveal mechanistic insights into these diseases and potentially lead to novel treatments.

## MATERIALS AND METHODS

### Animals

*Pum2* knockout (*Pum2*^*–/–*^) mice were created from an ESC line carrying a gene trap insertion (XE772) in the *Pum2* locus (*Pum2*^*XE772*^ or *Pum2*^*–/–*^ mice) [23]. In *Pum2*^*–/–*^ mice, no wildtype *Pum2* transcripts were detectable [23]. The inserted genetrap vector contains a lacZ reporter under the control of the *Pum2* promoter, hence lacZ expression could be used to track *Pum2* mRNA expression on a cellular level *in vivo*. In addition to the *Pum2*^*–/–*^ mice, *Pum2*^*E67*^ knockout mice were generated by removing exon 6 and exon 7 of *Pum2* [67]. Unlike *Pum2*^*XE772*^ which still produce a truncated and non-functional chimeric protein between PUM2 and LacZ, the brain tissue of *Pum2*^*E67*^ do not have any part of PUM2 protein left (Figure 5A), making this allele an unambiguous null allele of *Pum2*.

Adult mice (3–8 months older) from both sexes were selected for this study. Each group contained 4–6 animals for morphological and biochemical studies. The animals were maintained in a temperature-controlled facility at 40–50% relative humidity and 20–21° C average room temperature on a 12 h light/dark cycle. Food and water were available *ad libitum*. Mice were housed and bred in the barrier facility in the Northwestern University Center for Comparative Medicine and at Nanjing Medical University. All procedures involving animals were carried out in accordance with the Guide for the Care and Use of Laboratory Animals and were approved by both the Institutional Animal Care and Use Committees (ACUC) at Northwestern University and at Nanjing Medical University.

### X-gal staining

*Pum2*^*–/–*^ and wildtype littermates were perfused with 4% paraformaldehyde (PFA) and whole brains were dissected. The brains were in same fixation overnight at room temperature, transferred to 20% sucrose/PBS buffer for 24 hours, then embedded in Tissue-Tek O.C.T. (Electron Microscopy Sciences, Hatfield, PA, USA). Brains were cut in coronal section at 40 μm and mounted on slides. Sections were fixed in 2% PFA/PIPES buffer for 10 min on ice, and rinsed with Rinse Buffer (2 mM MgCl_2_/PBS) for 10 min on ice, then incubated in X-gal solution at 37° C overnight. Sections were rinsed with Rinse Buffer, 2% PFA/PIPES buffer, PBS + 2 mM MgCl_2,_ (each for 5 min) then counterstained with Neutral Red and dehydrated with ethanol and mounted with Permount. Slides were examined on a Leica MRT compound microscope (Leica Camera AG, Solms, Germany), and images were captured with the Nikon DXM1200 camera and native ACT-1 image software (Nikon Corporation, Tokyo, Japan).

### Dendritic number and spine density

Golgi staining was conducted using the Rapid Golgi Stain Kit (FD Neurotechnologies, Ellicott City, MD, USA) according to manufacturer’s instructions. Briefly, the brains were immediately removed and rinsed in 0.1 M phosphate buffer. Brains were immersed in a Golgi-Cox solution, replaced once after 12 hours of initial immersion, and stored at room temperature in darkness for 2–3 weeks. After the immersion period in the Golgi-Cox solution, brains were transferred to a cryoprotectant solution and stored at 4° C for at least 48 hours in the dark before sectioning. Brains were rapidly frozen with dry ice and cut with coronal plain at approximately 150 um thickness on a cryostat. The sections were transferred onto gelatin-coated slides and air dried at room temperature in the dark. After drying, sections were rinsed with distilled water and were subsequently stained in developing solution and dehydrated, cleared, and cover-slipped with Permount. Pyramidal cells from the CA1 layer of the dorsal hippocampus and from Layer IV of cortical sections directly superior to the dorsal hippocampus were compared across age-matched *Pum2*^*–/–*^ and WT mice. Dendritic spine density was measured in the on screen live view using the Nikon DXM1200 camera, allowing spines from multiple focal planes to be counted at 1000× magnification by a researcher blind to genotype and experimental conditions. At 5 neurons per section, a total of 15 neurons from 3 sections per animal were selected in corresponding rostral-caudal locations. Spines were counted along the first 15–30 µm of the first primary dendrite branching from the large apical dendrite. Only cells with clearly visible dendrites and easily identifiable secondary structures and soma were included, with the selection of cells otherwise randomized within the chosen location. To allow for comparison of data and determination of relative spine density, the totals were standardized to reflect the number of dendrites per 10 µm span. While it is known that manually counting the number of spines from Golgi-stained slides will lead to gross underestimations, using the same method across all animals allows for a comparison of relative number of spines among the animals.

Overall primary dendritic length and secondary branching were evaluated via Sholl analysis. 30 µm concentric rings were drawn around neurons selected using the same criteria as described above by a researcher blind to genotype and experimental conditions. Measurements were made at 400× magnification. Five neurons per section and a total of three sections per animal were captured with Nikon ACT-1 software and analyzed in Image J (NIH-Image 1.62, Bethesda, MD, USA) by a blinded observer. Total dendritic length and branch intersections represent the sum of the Sholl output up to 150 µm away from the soma. The total number of primary branches was also counted from the same area of the hippocampus and cortex.

### Synaptic density

For synaptic density analyzed by electron microscopy, animals were deeply anesthetized and perfused transcardially with 0.01M PBS containing heparin sodium for 2 min, followed by a 30 min perfusion with 2% paraformaldehyde, 2% glutaraldehyde, and 4% sucrose in 0.1M PBS. 250 µm sections were cut in the coronal plane using a vibratome. Fifteen sections encompassing the whole hippocampus were selected from each brain and were rinsed in cold 0.1 M PBS, treated with 2% OsO_4_ in 0.1M PBS for 90 min at 4° C, and rinsed again in 0.1 M PBS at room temperature. The sections were then dehydrated in a graded series of ethanol solutions, followed by propylene oxide, and left overnight in a 1:1 mixture of propylene oxide-Polybed 812 (Electron Microscopy Sciences, Hatfield, PA, USA). Finally, the sections were flat embedded in Polybed 812 in an oven at 60° C for 48–72 hours. From the 15 embedded sections, three representative sections included the dorsal, medial, and ventral hippocampus, and underlying cortices were selected for semi-thin and ultra-thin sectioning.

Selected embedded sections (250 µm thick) were trimmed and sectioned again using a Reichert Ultracut E Ultramicrotome (Austria). Semi-thin (1 µm) sections that included the hippocampus and underlying cortex were cut and stained with toluidine blue as reference sections for ultra-thin cutting. The sections were trimmed and ultra-cut. The thin (75–90 nm) sections containing the outer molecular layer of the dentate gyrus (dorsal blade) were mounted on 400-mesh grids (62 × 62 µm^2^; Electron Microscopy Sciences, Hatfield, PA, USA). The sections were stained using 3% uranyl acetate for 20 min followed by lead citrate for 5 min.

At low magnification under the electron microscope, the boundaries of the stratum radiatum of the CA1 subfield were identified according to their characteristic cellular structures. Six-ten photographs per section were taken systematically at 8000× magnification using alternate grid squares and 3 sections from each animal (18–30 photographs per animal), including the dorsal, medial, and ventral hippocampus, were assessed. Synapses were identified in electron micrographs that were enlarged photographically to a final magnification of 29,000×. Synapses were identified by the presence of synaptic vesicles and postsynaptic densities. All asymmetrical and symmetrical synapses were counted. The area of the unbiased counting frame was 247 µm^2^, the dissector height was 0.085 µm, and the dissector volume was 20.99 µm^3^. The latter value was used to calculate the density of synapses or degenerating axons (synapses or axons per unit volume) as the quotient of the mean number of synapses or axons counted per dissector, and the mean volume was examined using a JEOL 100 CX electron microscope (JEOL Ltd., Tokyo, Japan).

### Immunohistochemistry

Mouse brains were dissected and fixed overnight in Hartman’s fixative (Sigma) and processed for immunohistochemistry according to standard protocols [68]. Immunostaining for PUM2 was performed following citrate buffer antigen retrieval by incubation with anti-PUM2 (Bethyl Lab) primary antibody and detected using Biotin-Streptavidin HRP Detection Systems (ZSGB-BIO). For PUM2 and GLUR2 co-localization in the hippocampus, brains were fixed with 4% paraformaldehyde (PFA) and cryostat cross-sectioned at 40 um thickness. The sections containing the dorsal hippocampus were blocked for 20 min with 1% bovine serum albumin in PBS. Immunoreactivity was detected using a rabbit anti-PUM2 polyclonal antibody (Milipore, 1:200) and mouse- anti-GLUR2 monoclonal antibody. Visualization of the second marker was accomplished using species-specific secondary antibodies conjugated with cyamine dye (Cy3), fluorescein isothiocyanate (1:200, Jackson ImmunoResearch, West Grove, PA, USA), or Alexa 488 (1:100; Molecular Probes, Eugene, OR, USA) for confocal microscopy (Olympus Fluoview).

### Western blot

After mice were anesthetized and euthanized, the frontal cortex and hippocampus were quickly dissected on ice, snap-frozen, and stored at –70°C until biochemical analyses were conducted. The frozen brains were allowed to thaw on ice and were then homogenized in 5 volumes of ice-cold homogenization buffer (0.2% NP-40 and protease inhibitor in PBS buffer). Homogenates were centrifuged at 15,000 g for 20 minutes at 4° C, and the supernatant were used to measure protein levels in the brain (BCA method). 25 µg of each sample was further diluted in sample buffer (Bio-Rad, Hercules, CA, USA) and 15% polyacrylamide gels were used for SDS-PAGE. Proteins were transferred to PVDF membranes probed with primary antibodies against mouse PSD95 (1:1000 dilution) (Sigma-Aldrich, St. Louis, MO, USA); synaptophysin (1:500 dilution) (Cell Signaling Technology, Boston, MA, USA); glutamate receptor 2 (1:1000 dilution) (Millipore, Billerica, MA, USA); Rabbit PUM2 (Bethyl Laboratories, A300-202A; 1:500); mouse N-methyl-D-aspartate (NMDA) receptors 2A and 2B (1:500 dilution) (PhosphoSolutions, Aurora, CO, USA); phospho-cAMK2 (1:500) (Millipore); and β-actin (1:1000 dilution) (Santa Cruz Biotechnology, Santa Cruz, CA, USA), followed by horseradish peroxidase (HRP)-conjugated secondary antibody binding (1:20,000) (BD Diagnostic Systems, Sparks, MD, USA). Immunoreactive proteins were visualized using the enhanced chemiluminescence Western blot detection system (Thermo Fisher Scientific, Waltham, MA, USA). Light-emitting bands were detected with X-ray film (Thermo Fisher Scientific). Quantification of individual bands was performed using ImageJ software (NIH-Image 1.62) by plotting density. The relative concentrations of target protein in each sample were measured by comparing target protein band density to β-actin band density in the same well.

### Quantitative real-time PCR

Total RNA was isolated using Qiazol (Qiagen) and genomic DNA contamination was eliminated with TURBO DNase (Ambion). Reverse transcription of RNA was carried out with iScriptTM cDNA synthesis kit (Bio-Rad), according to the manufacturer’s protocol. Relative quantification of gene expression through the ΔΔC_q_ method was conducted with the Applied Biosystems 7300 Real Time PCR System (Applied Biosystems) using the iTaq SybrGreen Supermix with ROX (Bio-Rad). PCR results were normalized to the expression of actin.

### Immunoprecipitation

Hippocampi of wildtype or *Pum2*^*–/–*^ mice were extracted with PLB buffer (100 mM KCL, 5 mM MgCl2, 10 mM Hepes, pH 7.0, 0.5% Nonidet P-40, 1 mM Dithiothrectol (DTT), 100 units/ml RNase OUT (Invitrogen-cat# 10777-019), supplemented with RNAse inhibitors and protease inhibitors. The lysate was pre-cleared with 15 ug of rabbit Ig and 50 µl protein G/A sepharose, then the protein concentration was measured. Hippocampal lysates were immunoprecipitated using protein A Sepharose beads (Amersham Pharmacia Biotech) and pre-incubated with 30 ug of anti-PUM2 (Bethyl Lab). Protein A conjugated Sepharose beads without antibodies were used as negative controls. After immunoprecipitation the beads were extracted with acid phenol-CHCI_3_ (Ambion) to isolate RNA, followed by qRT-PCR using oligo dT (Promega) and amplification using mouse *Glur2* specific primers (*Glur2* specific primers forward: 5′GCCGAGGCGAAACGAATGA3′ reverse:5′ CACTCTCGATGCCATATACGTTG3′ and mouse actin primers forward: 5′TGACCCAGATCATGTTTGAG3′ reverse:5′GAGTCCATCACAATGCCTG3′)

### Dual fluorescent assay of PUM interaction with 3′UTR of *Glur2*

Three overlapping fragments from *Glur2* 3′UTR containing PBEs were subcloned into a Psi-check 2 plasmid (Promega) with XhoI and PmeI restriction enzymes (New England Lab). The 3′UTR fragments were amplified from brain cDNA using *Glur2* 3′UTR specific primers containing restriction enzyme sites for later cloning. The psi-check 2 plasmids were transfected in NIH3T3 cells in 48-well plates. After 24 hr, *Firefly* and *Renilla* luciferase expression were measured respectively using the Dual Luciferase Reporter Assay System (Promega) according to the manufacturer’s instructions in a Biotek Synergy 2 multi-mode Microplate Reader (Vermont, USA).

### Polysome fractionation experiment

Polysome profile analysis was carried out as previously described [69]. Six hippocampus tissue from adult mouse of each genotype were treated with ice-cold PBS containing 100 μg/ml cycloheximide and subsequently lysed in a polysome lysis buffer (100 mM KCl, 0.1%Triton X-100, 50 mM HEPES, 2 mM MgCl2, 10% glycerol, 100 μg/ml cycloheximide, 1 mM DTT, 20 unit/ml RNase Inhibitor(EDTA free) and 1 × cocktail). Lysates were loaded onto 20–50% (w/v) sucrose density gradients (10 mM Tris-HCl [pH 7.5], 5 mM MgCl2, 100 mM NaCl and 1 mM DTT) and centrifuged at 38,000 rpm for 2.5 hr at 4° C in a Beckman SW41 Ti rotor. Gradients were fractionated, and the absorbance at 254 nm was continuously recorded using Gradient Fractionator (BioComp, Canada).

### Data analysis

Two-sample comparisons were carried out using Student’s *t*-test (two-tailed), while multiple comparisons were made using one-way ANOVA followed by Tukey’s *post hoc* test. All data were presented as mean ± S.E.M., unless noted, and the limit for statistical significance was maintained at *P* value < 0.05. Values where *P* < 0.001 are regarded as highly significant.

## SUPPLEMENTARY MATERIALS FIGURES


