# Supplementary material for: Pumilio2 regulates synaptic plasticity via translational repression of synaptic receptors in mice
Source: Oncotarget. 2018 Jan 30;9(63):32134–48. doi: 10.18632/oncotarget.24345 (PMC6114944; doi:10.18632/oncotarget.24345)
Supplement: Supplementary file 1 [file oncotarget-09-32134-s001.pdf]

Pumilio2 regulates synaptic plasticity via translational repression of synaptic receptors in mice

SUPPLEMENTARY MATERIALS

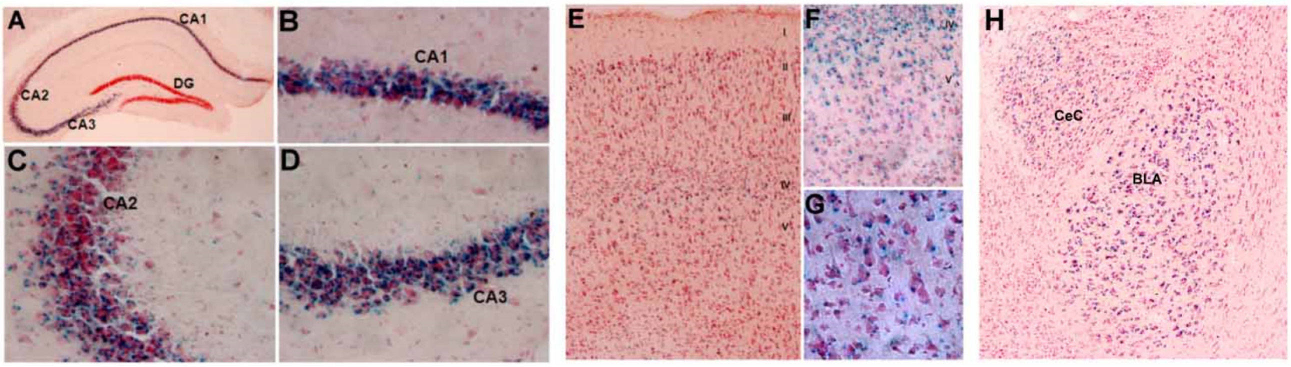

Supplementary Figure 1: Coronal sections of mouse brain from heterozygote *Pum2* gene trap mice *Pum2*^{XE772} was stained with X-gal. LacZ Positive cells staining blue color, indicative of *Pum2* mRNA expression, were seen in many brain regions of the mice, including the hippocampus (**A–D**), frontal cortex (**E–G**) and the amygdala (**H**). Nuclei stained red in the images).

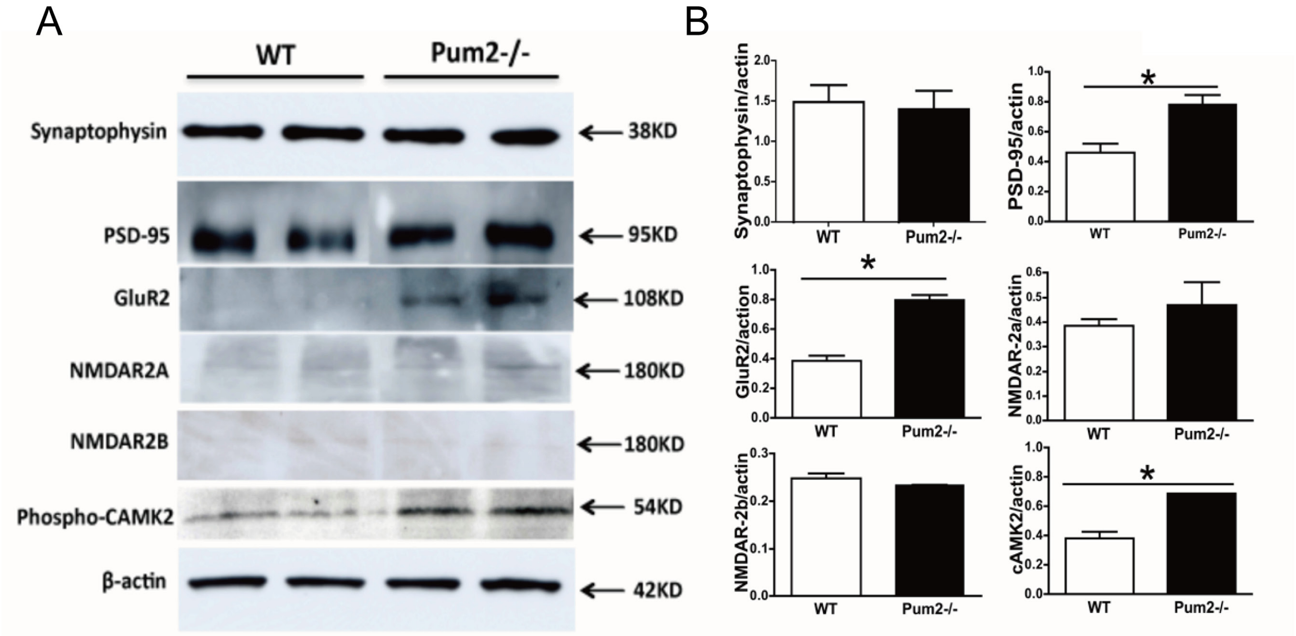

Supplementary Figure 2: Western blot analysis of synaptic related protein expression in the hippocampus in the absence of Pum2. (**A**) PSD95, GLUR2, and phospho-CAMK2 were significantly increased in *Pum2*$^{-/-}$ mice. (**B**) Western blot signal intensity was quantified showing significant increases in *Pum2*$^{-/-}$ mutant tissues for PSD-95, GLUR2, and CAMK2. *means $p < 0.05$. Data represent as mean ± SEM.

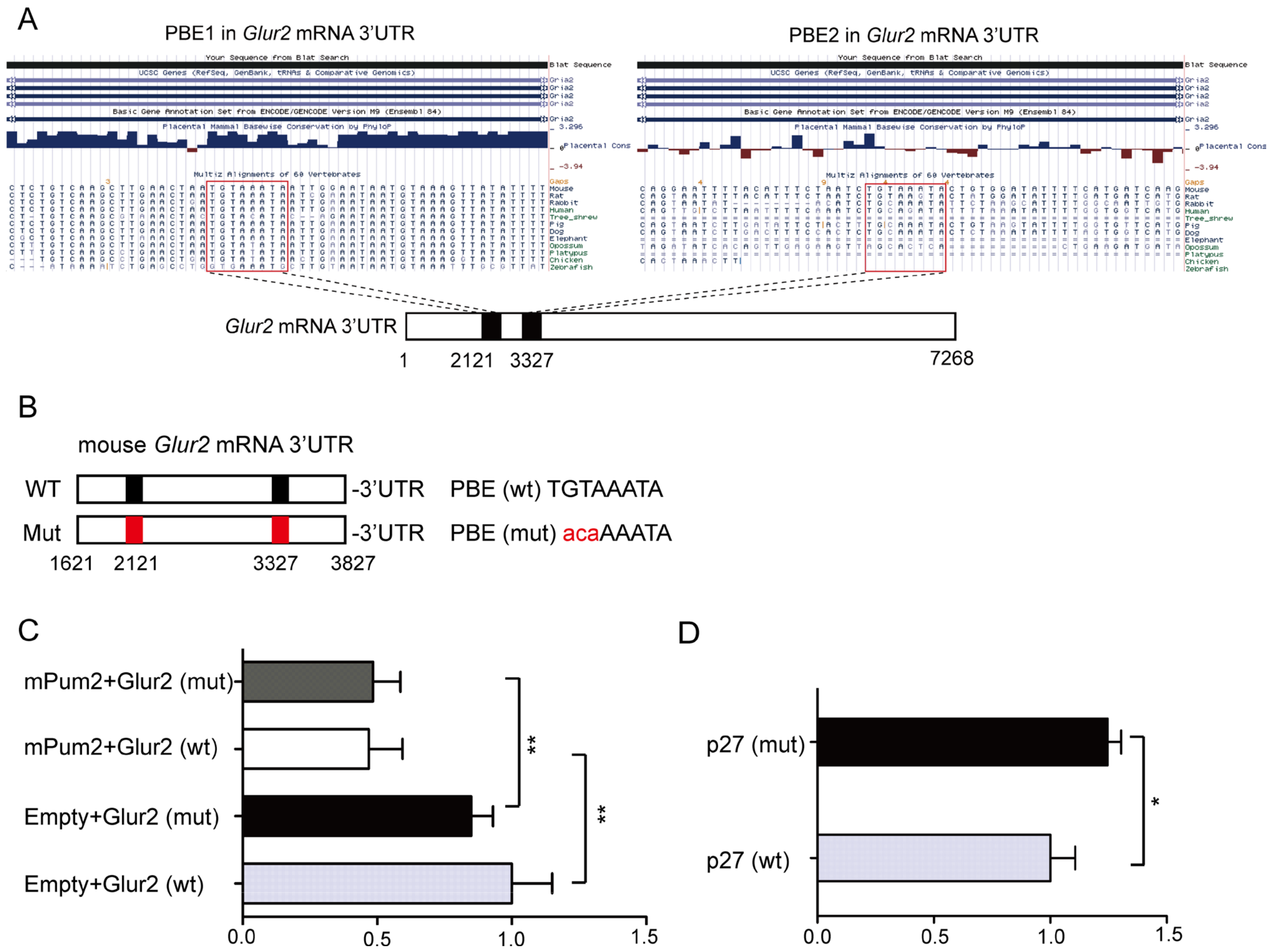

Supplementary Figure 3: Schematic illustration of mouse *Glur2* 3'UTR showing two putative PUM2 binding elements (red and black boxes) and their conservation in different species. (**A**) Schematic illustration of mouse *Glur2* 3'UTR showing two putative PUM2 binding elements (black boxes) and their conservation in different species. The numbers indicate positions of PUM2 PBE elements in the mouse *Glur2* 3'UTR. (**B)** Schematic representation of wildtype *Glur2* 3'UTR showing the location of PBE and mutated PBE in mutated UTR. (**C**) Luciferase assay performed as in B. Results of luciferase assay showing repression of PUM2 on wt 3' UTR and mutated 3'UTR of *Glur2*. (**D**) *p27* , as a known PUM target, serves as positive control of Luciferase assay.
